# Virtual healthcare solutions in heart failure: a literature review

**DOI:** 10.3389/fcvm.2023.1231000

**Published:** 2023-09-07

**Authors:** Keni Cheng-Siang Lee, Boris Breznen, Anastasia Ukhova, Seth Shay Martin, Friedrich Koehler

**Affiliations:** ^1^ General Medicines Global Business Unit, Sanofi, Paris, France; ^2^ Evidence Synthesis, Evidinno Outcomes Research Inc., Vancouver, BC, Canada; ^3^ General Medicines Global Business Unit, Sanofi, Moscow, Russia; ^4^ Ciccarone Center for the Prevention of Cardiovascular Disease, Johns Hopkins University School of Medicine, Baltimore, MD, United States; ^5^ Deutsches Herzzentrum der Charité (DHZC), Centre for Cardiovascular Telemedicine, Campus Charité Mitte, Berlin, Germany; ^6^ Division of Cardiology and Angiology, Charité – Universitätsmedizin Berlin, Corporate Member of Freie Universität Berlin and Humboldt-Universität zu Berlin, Berlin, Germany

**Keywords:** heart failure, virtual healthcare, telemedicine, eHealth, patient empowerment, self-care

## Abstract

The widespread adoption of mobile technologies offers an opportunity for a new approach to post-discharge care for patients with heart failure (HF). By enabling non-invasive remote monitoring and two-way, real-time communication between the clinic and home-based patients, as well as a host of other capabilities, mobile technologies have a potential to significantly improve remote patient care. This literature review summarizes clinical evidence related to virtual healthcare (VHC), defined as a care team + connected devices + a digital solution in post-release care of patients with HF. Searches were conducted on Embase (06/12/2020). A total of 171 studies were included for data extraction and evidence synthesis: 96 studies related to VHC efficacy, and 75 studies related to AI in HF. In addition, 15 publications were included from the search on studies scaling up VHC solutions in HF within the real-world setting. The most successful VHC interventions, as measured by the number of reported significant results, were those targeting reduction in rehospitalization rates. In terms of relative success rate, the two most effective interventions targeted patient self-care and all-cause hospital visits in their primary endpoint. Among the three categories of VHC identified in this review (telemonitoring, remote patient management, and patient self-empowerment) the integrated approach in remote patient management solutions performs the best in decreasing HF patients' re-admission rates and overall hospital visits. Given the increased amount of data generated by VHC technologies, artificial intelligence (AI) is being investigated as a tool to aid decision making in the context of primary diagnostics, identifying disease phenotypes, and predicting treatment outcomes. Currently, most AI algorithms are developed using data gathered in clinic and only a few studies deploy AI in the context of VHC. Most successes have been reported in predicting HF outcomes. Since the field of VHC in HF is relatively new and still in flux, this is not a typical systematic review capturing all published studies within this domain. Although the standard methodology for this type of reviews was followed, the nature of this review is qualitative. The main objective was to summarize the most promising results and identify potential research directions.

## 1. Introduction

Heart failure (HF) represents a growing social burden that significantly impacts the quality of life of individual patients and imposes escalating costs on healthcare systems (1). Recent data in the US show that an estimated 6.2 million people have HF, but the projections are worrisome since it is expected that by 2030 more than 8 million people will have this condition (2). The rise in HF incidence is not a problem unique to the US; globally, the absolute number of HF cases increased by 91% between 1990 and 2017 and reached 64.3 million (3).

Although the introduction of new drugs and devices led to significant progress in the treatment of HF in recent years, the mortality in HF patients remains high, reaching up to 65% at 5 years after diagnosis (4, 5). One reason for this is that only a small proportion of patients with HF achieve optimal doses of recommended HF therapy (6), and treatment adherence remains a persistent challenge in this population (7). In addition, HF typically evolves by bouts of hemodynamic deterioration, triggered by a vast number of factors leading to frequent and high-mortality hospitalizations (8). Therefore, the traditional in-clinic monitoring of HF patients is often inadequate to capture early signs of deterioration in time. Given that the earliest detectable changes in physiological measurements might occur prior to the onset of symptoms (6), timely detection of impending crisis is crucial to avoid hospitalization and premature death.

The widespread adoption of mobile technologies offers an opportunity for implementing solutions that can capture the early signs of cardiac decompression and provide timely intervention. Non-invasive remote monitoring and two-way, real-time communication between the clinic and home-based patients (9, 10), are already being tested in pilot trials in the HF population.

In the literature, these solutions are alternately referred to as telehealth, telemedicine, eHealth, mHealth, and several other creative names, reflecting lack of consensus on a proper terminology in the field. This review will adopt the term “virtual healthcare” (VHC), broadly defined as the remote delivery of healthcare via connected devices, mobile phones or tablets, and related internet technologies. The efficacy of VHC interventions is still in the early stages of investigations, with several studies reporting encouraging results in HF populations (11, 12), although questions regarding the comparative effectiveness of specific devices remain open (13).

While defining the objectives of this review, the authors noted that one neglected research topic is the challenge related to upscaling of the results of small-size clinical trials into real-world settings. In contrast to established procedures of real-world drug and medical device development, approval, and distribution, widespread utilization of VHC solutions poses additional challenges. The logistics of VHC deployment among the patients and caregivers require a much more active role of patients as well as an increased level of cooperation and shared decision making among healthcare professionals. In addition, the large amount of data gathered by mobile devices necessitate new approaches to data acquisition, storage, and analysis. Here, the adaptation of artificial intelligence algorithms (AI) will be necessary not only to process the real-time data inflow but also to aid in advanced diagnostics, personalization, and decision-making. These issues are largely neglected in existing clinical trials, where the focus is mainly on the comparative efficacy and utility of VHC interventions.

This review surveys the current state of VHC in HF with a focus on the practical aspect of VHC implementations and its challenges in real-world situations, summarizing existing evidence on methodology, efficacy, and integration into routine clinical settings.

## 2. Materials and methods

Standard methodology for conducting and reporting systematic reviews recommended by the Cochrane Collaboration's Handbook for Systematic Reviews of Interventions was adapted to conduct a literature review (14). The review surveyed VHC solutions in patients with HF with the research objective to describe and characterize the landscape of evidence of the past 5 years (2015–2020) of the approved and in-development virtual or mobile healthcare solutions, defined as a care team + connected devices + a digital solution (e.g., a smartphone app and/or wearable devices). The results were then summarized in a narrative form.

Using predefined search strategies, Embase was searched via the Ovid platform from inception until December 6th, 2020 (Supplementary Table S1). All abstracts identified by the search were reviewed by a single reviewer according to predefined, PICOS-framed eligibility criteria (Supplementary Table S2). All studies identified as eligible during title/abstract screening were then screened at the full-text stage by two reviewers. A Preferred Reporting Items for Systematic Reviews and Meta-Analyses (PRISMA) diagram was generated for complete transparency and reproducibility of the search and screening process (15).

A standardized data extraction table was generated to define the study characteristics, patient characteristics, intervention characteristics, and outcomes that were extracted from eligible studies. Quality control procedures were undertaken during data extraction to verify the accuracy and completeness of each collected data point.

An additional search targeting studies with keywords related to scaling up VHC solutions in HF within the real-world setting was performed on September 27th, 2021 to supplement the evidence base (Supplementary Table S3). The need for additional search arose *post hoc* after summarizing the main body of evidence. Evidence from this search was incorporated narratively into the results.

One of the results of reviewing the included studies and observing shared methodological patterns was identification of three distinct paradigms of VHC interventions. These three paradigms were distinguished by the clinical objectives targeted by the VHC interventions:

○ Telemonitoring: remote monitoring of cardiac and extra-cardiac variables with regular uploading and evaluation of the data at the centre in order to detect early signs of cardiac decompensation.
○ Remote patient management (RPM): an integrated healthcare solution that includes telemonitoring as its part, but it also provides a service platform for real-time interactive patient-clinician communications.
○ Self-care: solutions that empower patients in their independent decision-making process of sustaining health through disease education, symptom monitoring, treatment-seeking, and evaluating the effects of treatment.

The *post hoc* rationale for this classification and examples of each category are below:

• The telemonitoring studies used as a part of the intervention a device or a mobile app measuring clinical variable(s) with regularly scheduled automatic data uploads. Data uploads did not require the patient's involvement, and data evaluation was performed by medical staff at the health centre. A typical example is a study evaluating the efficacy of a device for remote monitoring of lung fluid by measuring dielectric properties of tissues (16). Measurements were transmitted via a cellular data link to a secured server for review by a health care professional using a dedicated web-based electronic data capture and viewing system.
• The RPM studies included, in addition to telemonitoring, direct regular interactive communication between the patient and the healthcare centre. The main purpose was to establish regular human-to-human contact and to offer medical advice, answering patients' questions, and provide encouragement. The communications channels usually included videoconferencing, phone calls, or dedicated websites.
• The self-care interventions were usually stand-alone apps (17) or self-contained devices such as an accelerometer (18) providing patients feedback and information about their condition and advice for behavioural changes to improve their health and well-being.

The AI in the context of this review is defined as machine-based data processing to achieve objectives that typically require human cognitive function (19). The studies reporting on the use of AI in HF can be broadly classified into three groups based on the purpose of the algorithms:

• Primary diagnostics: the algorithms are used to identify patients with HF among a wider population of patients either in primary care (20) or among hospitalized patients (21).
• Phenotype identification: the algorithms are used to identify different phenotypes within the primary diagnosis of HF by evaluating the associations of a variety of clinical parameters with pre-specified subgroups of patients (22–25). The word “phenotype” in the studies was used in broad sense and the identified phenotypes did not always correspond to traditional clinical phenotypes as understood by cardiologists. This was especially true for unsupervised learning algorithms (26–28) where the data set was comprised of large number of extracted features and the algorithm was trying to find clusters in hyperdimensional feature space. The resulting clusters were then interpreted *post hoc* by the researchers.
• Outcome prediction: this was the largest set of studies, where the algorithms were used to predict outcomes such as mortality and hospitalization risks (29–31), re-admission (32–34), tissue remodelling (35, 36), and a variety of other clinical outcomes (22, 37–39).

## 3. Results

A PRISMA flow diagram of the study selection procedure is presented in Supplementary Figure S1. A total of 171 studies were included for data extraction and evidence synthesis: 96 studies related to VHC efficacy, and 75 studies related to AI in HF. In addition, 15 publications were included from the search on studies scaling up VHC solutions in HF within the real-world setting (PRISMA flow diagram in Supplementary Figure S2).

Below is the summary of the results for the efficacy data set of VHC in HF. Table 1 shows the distribution of the study types for the 96 included studies.

**Table 1:** The number of included studies of each respective study type in the efficacy evidence base.

Study type	Number of included studies
Interventional studies (total):	62
Randomized controlled trials (RCT)	37
Prospective, non-randomized trials	7
Single-arm trials	17
Pooled comparative studies	1
Observational studies (total):	34
Prospective	10
Retrospective	15
Cross-sectional	9

The population sizes ranged from 10 patients (40, 41) to 3,449 patients in one study retrospectively analysing adherence to a telehealth program in US Veteran Administration centres (42). The mean population size in the included studies was 311 patients, and the median was 110 patients. This discrepancy is caused by the one outlier study, including 3,449 patients. The summary of study characteristics and population characteristics can be seen in supplementary materials (Supplementary Figures S3, S4).

The inclusion criteria for patient enrolment in most of the studies were based on the New York Heart Association (NYHA) functional classification (43). Seventy out of the 96 included studies used this classification for patient inclusion. Twenty-one of the 96 included studies enrolled only patients with reduced ejection fraction defined as less or equal to 40%. Forty studies enrolled a mixture of patients with both reduced and preserved ejection fractions. Thirty-five studies did not include information about ejection fraction (EF) status among the patients. Nineteen studies reported average EF of the enrolled population ranging from the minimum average EF of 21.5% (44) to maximum of 58% (45) with overall mean of 34.1%. Four studies enrolled patients with wearable cardioverter defibrillators which were used for remote data acquisition (44, 46–48).

The three VHC categories (telemonitoring, RPM, self-care) were used to compare clinical efficacy of the interventions. Table 2 shows the total number of studies and the number of RCTs within each of these categories. Both the telemonitoring and remote care studies used some form of remote data capture and upload. The types of patient data captured varied across the studies. The most commonly monitored data were self-reported patient symptoms (40, 49, 74, 75, 124), physical activity measured by accelerometers (18, 46, 125–127), body weight, blood pressure, and heart rate (50, 76–79), data transmitted by cardioverter defibrillators (44, 46–48, 76), and others.

**Table 2:** The number of studies in the VHC intervention categories.

Intervention category	Number of studies (all)	Number of studies (RCTs)
Telemonitoring	30 (16, 44, 46–73)	14 (46, 49, 50, 52, 54–56, 59–61, 65, 70–72)
RPM	53 (41, 42, 45, 74–122)	21 (45, 74, 75, 80–82, 86, 88, 91–93, 97, 98, 102, 107, 112, 113, 115–117, 123)
Self-care	13 (17, 18, 40, 124–133)	2 (130, 133)

### 3.1. Outcomes targeted by VHC interventions

There were 27 distinct primary outcomes reported across all included studies. The top ten most reported primary outcomes, together with the number of studies reporting significant results in the respective primary outcome, are summarized in Table 3. The data for all included studies, as well as the subset of data pertaining only to RCTs, are shown.

**Table 3:** Summary of primary outcomes reported across the included studies.

Primary outcome	Number of studies reporting the outcome	Number of significant results	Number of RCTs reporting the outcome	Number of significant results (RCTs only)
Mortality (49, 50, 63, 64, 71, 102, 116)	7	2	5	0
Re-hospitalization (16, 57, 58, 65, 67, 72, 87–90, 105, 108, 109, 127, 129)	15	8	3	1
ER visits (99, 112, 123)	3	1	2	1
Hospital visits (all-cause) (48, 60, 94–96)	5	4	1	1
Medication adherence (42, 61, 76, 77, 104, 106, 117, 126, 132, 134, 134)	12	3	4	2
Quality of life (70, 73, 110, 113, 114)	5	2	2	2
Depression (82, 107)	2	1	2	1
Self-care (59, 91–93, 111, 129, 130)	7	6	5	5
Patient experience (17, 18, 40, 56, 62, 63, 68, 78, 84–86, 103, 119, 120, 122, 124, 130)	17	0	2	0
Other (44–47, 51–55, 66, 74, 75, 80, 81, 83, 98, 100, 101, 115, 121, 125, 128, 131)	23	14	11	6

Medication adherence was generally reported as patients following the prescription schedule for drug taking for a significant fraction (usually >80%) of the study duration. Quality of life was measured using HF-specific questionnaires such as The Minnesota Living with Heart Failure Questionnaire or Kansans City Cardiomyopathy Questionnaire. Depression was evaluated using 9-item Patient Health Questionnaire. Self-care is a growing area of interest in all chronic diseases, and it was defined (with a few variations) as a process of maintaining health through health-promoting practices and by managing illness (e.g., by exercising, weight monitoring, taking medication, and seeking a health care provider when symptoms are deteriorating).

In the “Other” category, the most reported primary outcome was based on the series of publications related to the TIM-HF and TIM-HF2 trials (80–83). The primary outcome in this series was “the percentage of days lost due to unplanned cardiovascular hospital admissions and all-cause mortality”. In addition, the category “Other” also includes outcomes such as “cardiac acoustic biomarkers” (47), composite outcomes (51–54, 125), non-fatal HF episode (45), general health status (55, 74), functional capacity (128), and other unique endpoints.

The most frequently investigated primary outcome across all studies was the patient experience, loosely defined as “usability” (56, 84), “satisfaction/acceptability” (85, 124), “goal attainment or life satisfaction” (17), and variations thereof. However, most of these studies were non-comparative by design and therefore they described the outcome in a single arm. The two RCTs investigating patient experience (56, 86) reported high levels of patient satisfaction in both arms (with and without VHC), with no significant differences observed. The majority of the studies used the RPM paradigm (9/17, 53%), followed by telemonitoring (4/17, 23.5%), and self-care (4/17, 23.5%).

### 3.2. VHC interventions efficacy

The most successful VHC interventions, as measured by the number of reported significant results, were those targeting reduction in rehospitalization rates. Eight (16, 57, 58, 87–90, 127) out of 15 (53%) studies reported significant reduction in rates of rehospitalizations, including one RCT (88). The majority of the interventions targeting rehospitalization rates used RPM approach (8/15, 53%), followed by telemonitoring (6/15, 40%), and self-care (1/15, 7%).

In terms of relative success rate, the two most effective interventions targeted patient self-care and all-cause hospital visits in their primary endpoint. Six (59, 91–93, 129, 130) out of seven (85.7%) studies targeting improved self-care reported significant results. Interestingly, even for self-care primary endpoints, the most common intervention paradigm was RPM (4/7, 57.2%), followed by self-care paradigm (2/7, 28.5%) and telemonitoring (1/7, 14.3%). Four (60, 94–96) out of five (80%) studies reported significant improvements in reducing all-cause hospital visits in the HF population. Three out of the five studies (60%) in this category used the RPM paradigm, and two (40%) used telemonitoring.

Patient adherence as the primary endpoint was investigated in 12 studies, four of which were RCTs (61, 84, 133, 134). Two of the four RCTs reported significant improvement due to the VHC intervention (133, 134). In both cases the remote care model of intervention was used, with healthcare professionals aiding patients in adopting the technologies into their everyday routines. Both studies noted increased self-awareness of patients when managing their condition.

### 3.3. Artificial intelligence in HF

The total of 75 studies reporting on the AI algorithms in HF were included in the evidence base. Table 4 shows the distribution of the studies across the three categories described in the Methods section, together with the validation status. Most studies used internal validation of the algorithms either by splitting data into a training set and validation set and re-sampling the sets 5–10 times for cross-validation (30, 135–137) or leave-one-out (76, 138, 139) method. The data used for training and validation of the algorithms came from a variety of sources such as clinical trial database (30, 138), electronic health records (34, 140–142), or internal institutional databases (143, 144).

**Table 4:** Distribution of studies on AI in HF and their validation status.

Category	Total number of studies	Number of validated studies
Primary diagnostics	10	9
Phenotype identification	14	8
Outcome prediction	51	47

Currently, the majority of AI studies are using data gathered by routine in-clinic laboratory procedures such as patient characteristics (37), cardiovascular MRI (145), ECG (146) and others. The outputs of the algorithms can be classified into three general domains (see the Methods section):

• Primary diagnostics algorithms are being developed with the purpose to use readily available clinical data to identify patients with heart failure while in the hospital. Input data are gathered from electronic health records (20, 21, 141) or internal hospital databases (147, 148). Outputs of the algorithms are aimed at helping clinicians in correct diagnosis of HF in patients admitted for a variety of cardiac conditions.
• Phenotype identification algorithms are used to identify sub-groups of HF patients either using a pre-defined classification or using clustering methods to discover hidden groupings within the data sets. In the first case, algorithms are identifying phenotypes such as HF patients with cardiac amyloidosis relying on routinely determined laboratory parameters (22), diagnosing PLN p.Arg14del cardiomyopathy using ECG (23), or identifying responders to cardiac resynchronization therapy using 2D echocardiography data (24). Novel sub-groups discovered in studies using clustering algorithms included three distinct phenotypes in patients with HFpEF that may respond differently to treatments or interventions (26), six HFpEF phenotypes, for which significant differences in the prevalence of concomitant atrial fibrillation, anaemia and kidney disease were observed (27), and four subgroups in patients with hypertrophic cardiomyopathy with distinct ECG features leading to a novel risk stratification (28).
• Outcome prediction was the largest group of studies. The outcomes of interest varied across the studies but most of the algorithms focused on predicting treatment responses in terms of mortality (30, 31), re-hospitalization (149, 150), adverse events (39) and other related outcomes.

Out of the 75 papers on AI in HF, only eight were set in the context of VHC where the data was acquired remotely:

• Primary diagnostics was the goal of one study (151) that investigated whether wristband data can be used to predict a diagnosis of HF in a cohort of 97 monitored cardiac inpatients. The AI algorithm performed best when the wearable data was combined with demographics, medical history, and vital signs. The achieved discrimination defined by the test area under the curve (AUC) was 0.87 with the specificity of 72% and sensitivity of 90% (151).
• Phenotype identification was investigated in one study (152). The study explored the use of unsupervised machine learning to identify subgroups of patients with HF who used telehealth services in the home health setting. The study identified patterns of association between (1) mental health status, pulmonary disorders, and obesity and (2) healthcare utilization for patients with heart failure who used telehealth in the home health setting (152).
• Outcome prediction was investigated in the remaining six studies (76, 149, 150, 153–155). Two studies used retrospective data sets to predict clinical outcomes: one study analysed data from the Telemonitoring to Improve Heart Failure Outcomes trial to predict readmissions in patients with HF (149), and one study developed a model for cardiomyopathy prediction (154). Four clinical trials in this group incorporated AI and VHC prospectively into the study design. One study (150) examined the performance of a personalized analytical platform using continuous data streams to predict rehospitalization after HF admission (LINK-HF study). Participants were monitored for up to 3 months using a disposable multisensor patch placed on the chest that recorded physiological data. Data were uploaded continuously via smartphone to a cloud analytics platform. Machine learning was used to design a prognostic algorithm to detect HF exacerbation. The cloud-based analytics platform used a general machine learning method of similarity-based modelling to analyse collected data. The algorithm used a 1-minute trim-mean (10%) heart rate, respiratory rate, a cumulative gross activity, and posture as inputs. The platform was able to detect precursors of hospitalization for HF exacerbation with 76%–88% sensitivity and 85% specificity. The median time between initial alert and readmission was 6.5 (4.2–13.7) days (150). One study developed and validated an algorithm to predict the occurrence of ventricular tachyarrhythmia in HF patients with implantable-cardioverter defibrillator (153). The algorithm used heart rate variability data and machine learning to automatically predict ventricular arrhythmia. The algorithm achieved performance quantified by AUC of 0.81 for 5-minute prediction and mean AUC of 0.87–0.88 for 10 s prediction (153). One study (76) investigated whether certain user characteristics (i.e., personal and clinical variables) predict the use of remote monitoring systems (RMS) using advanced machine learning software algorithms in patients with HF. The data support that RMS use was higher in patients who did not receive care from a healthcare provider with HF specialty. The study findings also showed that participants who had an internal cardioverter defibrillator were more likely to use the RMS (76). One study (155) tested a telephone intervention algorithm for monitoring ventricular assist devices (VAD) in outpatients with HF. A structured inquiry was used to gather information on pump parameters, vitals, and symptoms which was then electronically categorized by an algorithm into five levels of severity. Propensity-adjusted 2-year survival (89% vs. 57%, *P* = 0.027) was significantly higher for the telephone intervention group compared to standard of care group (155).

### 3.4. Real-world scalability of VHC in HF

The challenges of scaling up VHC solutions to real-world implementation have been mostly neglected in the pilot clinical trials and only addressed in a handful of studies. Hovland-Tanneryd et al. (94) published their experience of validation of a home-based tool for HF patients, previously tested in an RCT, in a cohort in a primary care setting in a clinical controlled trial. The aim was to compare the RCT findings to the more pragmatic design of a validation project in primary care. Data from both trials were analysed with respect to HF-related in-hospital days, self-care behaviour, and system adherence during a 6-month intervention. The results in both settings were similar in terms of the risk ratio of in-hospital days (RCT RR = 0.72, Clinic RR = 0.67), and the pooled data set showed improvement in self-care by 27%, with median adherence of 94% (94).

Several studies focused on patient experience and barriers to acceptance in a wider population of HF patients (156–160). The most common themes explored were regimen complexity, forgetfulness, difficulty coping with side effects (159), convenience (157), trust, perceived risk (160), and added responsibility for nurses and caregivers (158). Elderly patients with HF face additional barriers to adopting VHC. A study by Cajita et al. found that older adults do not base their intention to use mHealth solely on perceived ease of use and perceived usefulness. Instead, the following themes emerged from the content analysis: facilitators included previous experience with mobile technology, willingness to learn mHealth, ease of use, presence of useful features, adequate training, free equipment, and doctor's recommendation (156).

The emergence of COVID-19 accelerated the adoption of VHC in the real-world management of HF patients (161, 162). It also revealed the challenges of transitioning the care delivery and administrative organization to conform to a new healthcare environment while still providing high-quality care. Sayer et al. (163) described the experience of a large tertiary HF program in widespread adoption of telehealth, restructuring outpatient care, initiating a shared clinic model, and introducing a comprehensive remote monitoring program to manage patients with HF and heart transplants. The transition employed a high-intensity telehealth approach, centralized monitoring and intervention program, and the development of video conference-based support groups. It also required a collaborative approach, with contributions from dedicated teams of inpatient clinicians, outpatient clinicians, and administrative staff (163).

## 4. Discussion

The technologies used in VHC solutions are relatively new, and consequently, their fit into the existing models of HF patient care is still being explored in a variety of settings. When considering the results reported in the reviewed studies, the challenge is in translating them into the real-world clinical practice. Unfortunately, few studies addressed the issue of scalability of the VHC solutions and potential issues that may impede their acceptance. In order to carefully assess the available pathways towards the wide deployment of VHC in HF populations one need to start by looking at what works in the context of clinical trials and then try to identify potential roadblocks in the scaling-up those solutions.

**Figure 1:**
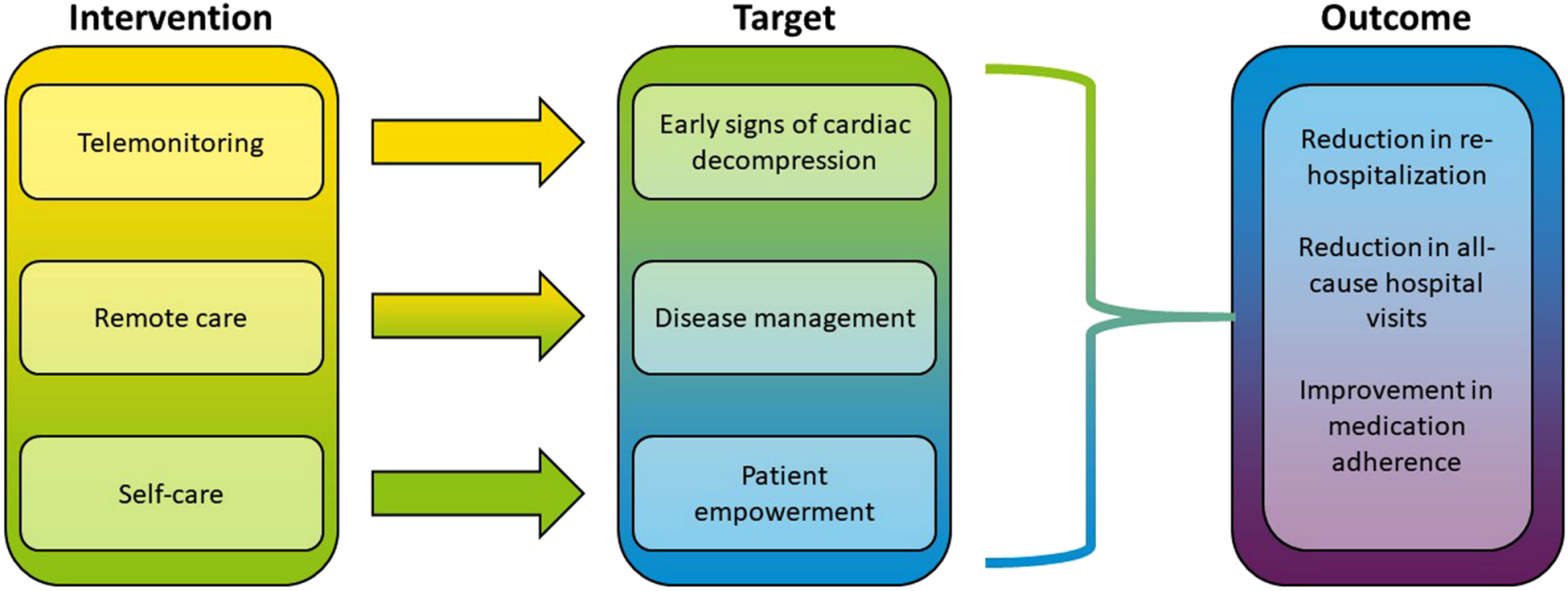
Summary view of the virtual healthcare intervention paradigm in heart failure patients.

From the standpoint of treatment efficacy, the RPM approach seems to be most effective among the three intervention categories (Figure 1). When looking at the endpoints where VHC showed the most success (rehospitalization, self-care, all-cause hospital visits), the majority of interventions used RPM paradigm. The multi-component intervention included telemonitoring devices with the daily upload of clinical measurements as well as communications with patients' general physician and cardiologist (80, 81). A similar approach integrating automated data monitoring with personalized communications was found in a majority of the studies (see Table 2). The RPM intervention could be as simple as a tool to encourage HF patients to work collaboratively with their clinicians to “make one positive change” in medication regimen (97), or a fully integrated system including a multidisciplinary care team consisting of a nurse coordinator, cardiologist, psychiatrist, and primary care physician, home telemonitoring, patient self-management support, and screening and treatment for comorbid depression (74). RPM was also used to interactively guide physical exercise in HF patients (94, 98) or to organize post-discharge remote visits and consultations (45).

The telemonitoring paradigm relies mostly on automatic data upload with limited patient involvement. Direct medical intervention is only triggered when the uploaded data cross predefined threshold signalling and impending crisis. The data upload can contain clinical variables such as lung fluid measures (16), medication taking (60, 62), vital signs (including, in some cases ECG) (50–52, 63, 64, 99), and self-reporting (55). A special case of remote monitoring concerns wearable cardioverter defibrillators, where the intervention is in part automated and implemented in the software of the wearable device (44, 46–48, 76). Historically, this paradigm pioneered the use of wearable devices and the concept of remote monitoring, however it is currently being integrated as part of the RPM model.

The concept of self-care in VHC includes a variety of approaches to patient empowerment, such as improving self-reported symptoms via mHealth apps (131), engaging the elderly patient population in technology-guided self-care (17, 124, 126, 132), and increasing self-awareness when managing patients' condition (130, 133).

There are several caveats to be considered when trying to generalize the results of the reviewed clinical trials into general practice. Most published studies are conducted in a controlled environment of research hospitals with relatively small groups of HF patients. The efficacy claims of the VHC interventions are valid within the pre-selected HF populations and limited follow-up of the studies. How will these results translate into a standard of care that can be applied to a variety of clinical settings is an open question. Even within the context of controlled studies, some results cast doubt on the efficacy of VHC interventions. For example, VHC interventions seem to have no effect on reducing all-cause mortality. Out of the five RCTs investigating this outcome none achieved significant improvement. Reasons are currently unclear and this issue merits further investigation. An interesting hypothesis to consider is the importance of direct human-to-human interactions aided by technology as reflected in the RPM paradigm. The number of significant results achieved by integrated solutions as compared to purely technology-based interventions may be, in part, explained by the presence of the human component. The technology is best utilized in support of shared decision-making by providing timely information and opening new channels for communications between patients and their care team. The main challenge going forward is to maintain this advantage in large-scale settings of existing healthcare systems.

With a few exceptions, the study durations did not extend beyond a 1-year follow-up. Given that the average lifespan of patients discharged from hospital with HF is 5.5 years (164), extending up to 19.5 years in a younger, low-risk population, the lack of long-term studies in this field represents an unmet need. In addition, HF patient populations span generations of patients starting from middle-aged “Gen-X”, through baby-boomers, to geriatric patients (see Supplementary Figure S4). Technology acceptance varies greatly among generations, hence a personalized approach is needed to achieve desired utilization. Here the studies analysing user experiences and barriers to acceptance are particularly valuable. For example, Woo et al. 2018 analysed factors facilitating/hindering the acceptance of VHC services in HF patients (165). Using a modified Unified Theory of Acceptance Use of Technology, the authors identified several factors associated with HF patients' initiation or use of VHC services in a home setting, some of which are relatively straightforward to implement (e.g., high-risk drugs education by visiting nurses).

One technological challenge to the general adaptation of VHC solutions is the large amount of clinical data the devices can collect and store. Particularly in the context of general use, this can be overwhelming, and clinicians will need assistance with the analysis and interpretation of the patient data. A promising development is the introduction of AI algorithms targeting the diagnosis, phenotyping, and prognosis in HF. The utility of using AI in diagnosing HF may be in aiding the clinician in deciding ambiguous cases (i.e., being a supporting factor to the clinical judgement of the physician). For example, an existing algorithm using a simple wristband achieved 74% accuracy (151) in diagnosing chronic HF (present/absent) from data of cardiology patients undergoing bedside monitoring. More sophisticated algorithms using better data sources (e.g., ECG) may be able to achieve greater diagnostic accuracy. Similarly, algorithms detecting sub-groups of HF patients using a collection of remote data (152) can identify clusters of patients that display different patterns of comorbidities and healthcare outcomes. This information can be then used to tailor the VHC intervention to address specific challenges facing the identified sub-groups. The limitations of AI are mostly in the academic nature of the algorithms with limited availability of these technologies where they are mostly needed—in real-world practice. To achieve a larger utilization, several barriers need to be overcome, such as gaining the trust in the algorithms by clinicians (by illustrating the physiology behind the algorithms), integrating the algorithms with the current workflow, resolving problems with privacy and data sharing, and providing adequate technical support in the field.

The topic of VHC in HF has been subject to several recent systematic literature reviews (6, 8, 162, 166). In this review, the intent was to highlight the comparative efficacy of the three identified categories of interventions: telemonitoring, RPM, and self-care. While summarizing the evidence, the limitations of the included clinical studies prompted additional research, including the questions of AI utilization and the real-world scaling of the results reported by the published studies. One of the findings in this review was that studies leveraging the synergy between AI and VHC in the HF population are limited in numbers. This seems like a potential area for future research. In particular, the AI algorithms can bring expert-level decision support for diagnostics and risk prediction to non-specialist general practitioners and facilitate the scaling up of the VHC solutions into real-world settings. A large-scale study of a distributed AI algorithms aiding clinicians in HF diagnostics and risk evaluations across multitude of real-world clinics could expose the barriers and challenges to AI acceptance.

Additional promising areas of future research include the apparent lack of efficacy of VHC intervention in reducing all-cause mortality in the HF population, long-term adherence and efficacy of VHC with follow-up spanning several years, and age-specific approach to technology adaptation with focus on the elderly HF patients. Additionally, given the relative novelty of these interventions and lack of experience in utilizing them in wider clinical settings, focus on education and transfer of knowledge may help in improving their acceptance and consequently their efficacy. This is particularly true in developing countries where the distances are large, and the number of healthcare professionals are limited.

### 4.1. Limitations

This is not a typical systematic review capturing all published studies within this domain. Although the standard methodology for this type of reviews was followed, the nature of this review is qualitative. Our intention was to provide a narrative summary of the most relevant findings related to the stated objectives of the study. The quantitative information provided here is selected based on the representativeness of the data without providing additional statistical analyses. The choice of narrative summary instead of the standard meta-analysis was made for several reasons. First, digital interventions in HF are heterogeneous both in the ways intervention is delivered and, in the variety of targeted outcomes. Unlike drug clinical trials where the methods of delivery and the measured outcomes are relatively well understood, for VHC interventions neither is true. How to deliver the intervention (e.g., text messages, phone-calls, video conferencing), frequency of the communications (e.g., daily, weekly, on-demand), and specific content of the communication are still being investigated. Methods of collecting clinical data (what to collect and how) and their upload are far from standardized. Targeted outcomes vary from easily quantifiable (mortality, re-hospitalization) to more ambiguous (patient experiences). Therefore, comparing the results using meta-analysis would require adopting many assumptions about the homogeneity of the included clinical trials. A narrative summary allows for a more qualitative description of the respective interventions although it lacks the rigor of quantitative meta-analysis and definite conclusions are more difficult to draw. However, given the exploratory nature of the clinical trials in VHC, a narrative summary may provide a useful way to survey the current state of the field.

## 5. Conclusions

The main finding of this review is that the promise of VHC in HF has been established in some studies but not all interventions reached their desired outcomes. The solutions with the most consistent positive impact on clinical outcomes are those that address already existing unmet needs such as early detection of warning signs of cardiac decompensation, easy patient access to the healthcare team, convenient access to relevant educational materials, and encouragement of healthy lifestyle. On the other hand, reducing mortality seems to be quite challenging and little progress has been achieved in boosting patient adherence. Artificial intelligence has a potential in interpretation of large data sets, but it is largely confined to academic institutions. Overall, while research studies administered by high-end academic institutions show some success, scaling the VHC intervention to general clinical practice remains a challenge.
