# Supplementary material for: Virtual healthcare solutions in heart failure: a literature review
Source: Front Cardiovasc Med. 2023 Sep 7;10:1231000. doi: 10.3389/fcvm.2023.1231000 (PMC10513031; doi:10.3389/fcvm.2023.1231000)
Supplement: Supplementary file 1 [file Table1.docx]

Supplementary Material

Virtual Healthcare Solutions in Heart Failure: A Literature Review

Keni CS Lee^*^, Boris Breznen, Anastasia Ukhova, Seth S. Martin, Friedrich Koehler

*** Correspondence:** Dr Keni Cheng-Siang Lee, CMD, PhD: [Keni.Lee@Sanofi.com](mailto:Keni.Lee@Sanofi.com)

# Supplementary Figures

#

# Supplementary Figure 1. PRISMA Diagram


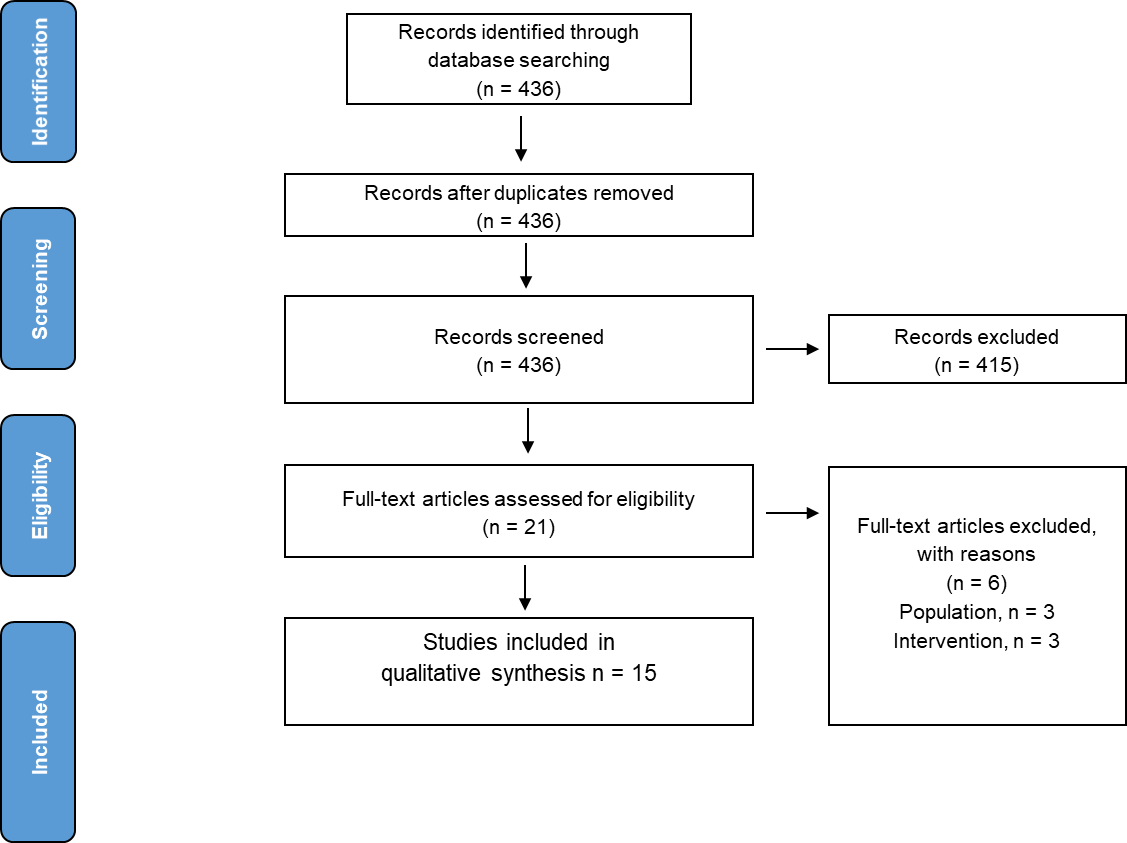


Supplementary Figure 2. PRISMA flow diagram for scaling up in the real-world setting.


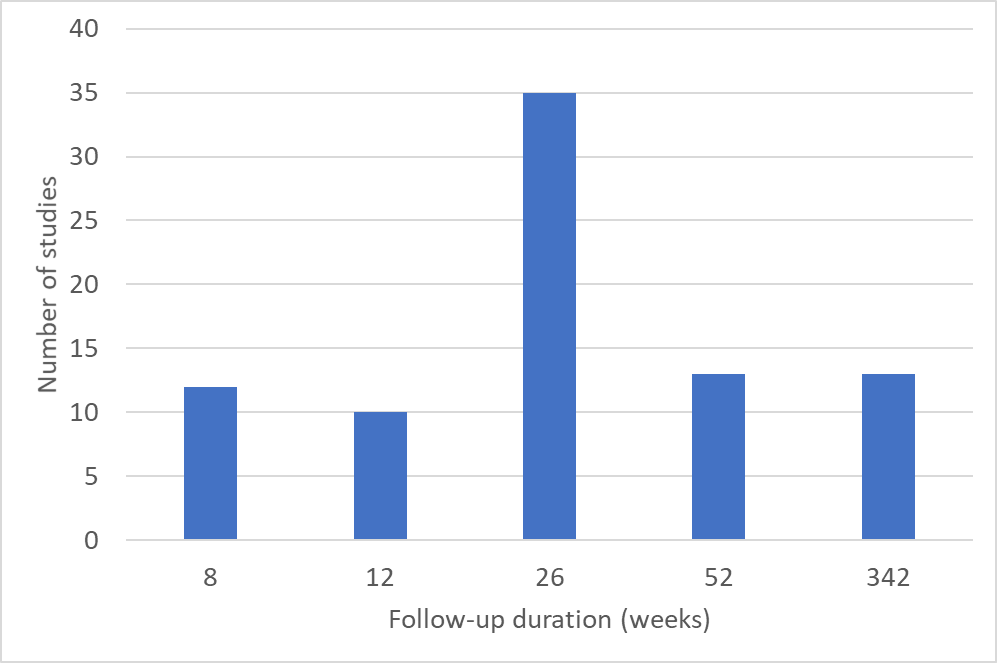


Supplementary Figure 3. Distribution of the mean follow-up durations.

**
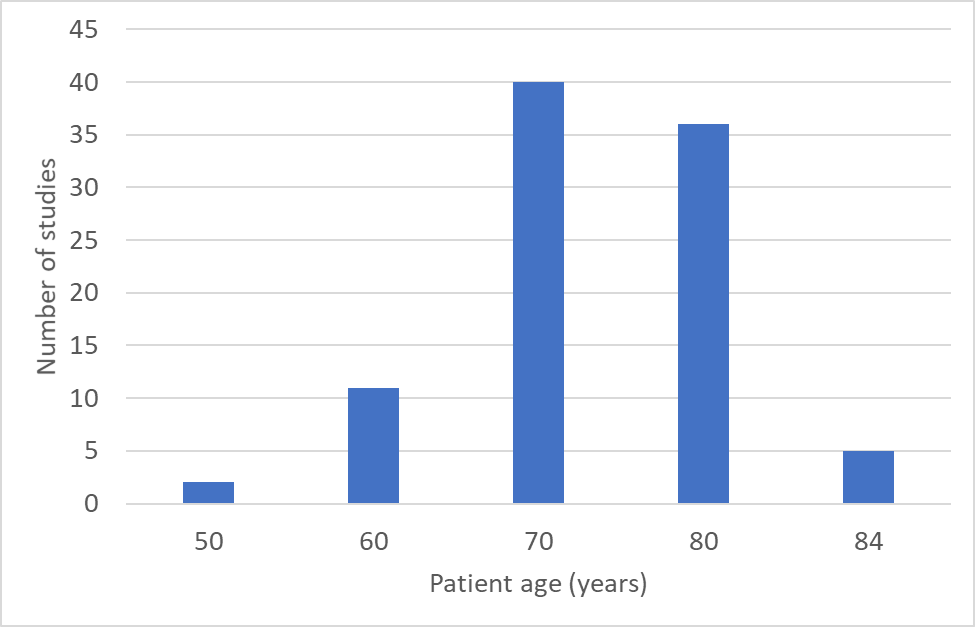
**

Supplementary Figure 4. Distribution of mean age across the included studies.

# Supplementary Tables

**Supplementary Table 1. Search strategy for Embase (via Ovid).**

| **Database: Embase**  **Segment used: Embase 1974 to 2020 December 4** | | |
| --- | --- | --- |
| **Search executed: December 6^th^, 2020** | | |
| **#** | **String** | **Hits** |
| 1 | exp heart failure/ | 516283 |
| 2 | exp Telecommunications/ or exp Telemedicine/ | 77552 |
| 3 | Telehealth.mp. | 12240 |
| 4 | (mhealth or ehealth).mp. | 8812 |
| 5 | (wearable* or gadget* or sensor* or smart* or "digital" or "machine" or ("mobile" adj2 (technolog* or "app" or "apps")) or electronic* or tablet* or accelerometer* or gyroscope* or tracker or (("speech" or linguistic* or language*) adj2 (analys?s or "recognition" or monitor* or pattern*))).ti,ab. | 1171207 |
| 6 | or/2-5 | 1237237 |
| 7 | 1 and 6 | 15205 |
| 8 | (book or chapter or editorial or erratum or letter or note or short survey or tombstone or comment or conference or conference abstract or conference review).pt. | 7996519 |
| 9 | Case Study/ | 74242 |
| 10 | case report.tw. | 433314 |
| 11 | (exp animal/ or nonhuman/) not exp human/ | 6539055 |
| 12 | or/8-11 | 14190776 |
| 13 | 7 not 12 | 7286 |
| 14 | limit 13 to (adult <18 to 64 years> or aged <65+ years>) | 3900 |
| 15 | limit 14 to yr="2015 -Current" | 2407 |

**Supplementary Table 2. PICOS eligibility criteria.**

| **PICO item** | **Inclusion criteria** | **Exclusion criteria** |
| --- | --- | --- |
| **Population** | Patients diagnosed with heart failure (HF) including but not limited to HF with reduced/preserved ejection fraction, right-sided HF, and congestive HF | N/A |
| **Interventions** | Virtual or mobile (mHealth) healthcare solutions including but not limited to mobile apps, wearable devices, telemedicine, text messaging, web-based communications, and personal computer-based solutions. | N/A |
| **Comparators** | Any or none | N/A |
| **Outcomes** | Efficacy/effectiveness outcomes:   - Physical activity/exercise - Blood pressure changes - Weight loss/maintenance - Adherence to medications - Smoking cessation - Healthy diet (salt intake, alcohol consumption, caffeine consumption, fruits/vegetables consumption, red meat consumption) - Stress management - Sleep patterns/disturbances - Emergency access to healthcare including unplanned physician visits, rehospitalizations, and emergency calls - Change from baseline measures (including 6MWT, left ventricular ejection fraction, frequency of acute care visits, readmissions) - Adherence to healthy lifestyle and behavioural changes - Psychological well-being - Acceptance/usability of intervention - Patient satisfaction - Barriers to acceptance and unmet needs   Safety Outcomes (including but not limited to):   - Total adverse events, device-related adverse events, serious adverse events, adverse events leading to discontinuation of study, major adverse cardiac events including not limited to: cardiovascular death, myocardial infarction, stroke, hospitalization (all reasons), - Increase of existing diuretic dose or addition of a new diuretic due to fluid retention - Hyperkalemia - Heart failure measures (heart failure progression, B-type natriuretic peptide biomarker change)   Health Related Quality of Life Outcomes   - Patient reported and Health Related Quality of Life (including but not limited to; EQ-5D, EQ-VAS, HeartQoL, MacNew, QLMI, MLHFQ) - Work-related productivity issues - Social engagement | N/A |
| **Study design** | - Randomized Controlled Trials (RCTs) - Non-randomized clinical trials - Observational studies - Cohort studies (retrospective and prospective) - Case-control studies - Cross-sectional studies - Systematic literature reviews | - Notes - Letters - Editorials - Comments - Case reports/series |
| **Additional criteria (limits)** | | |
| **Timing** | None | |
| **Setting** | None | |
| **Language** | English | |
| **Publication date limit** | 2015 onward | |

**Supplementary Table 3. Search strategy for Embase (via Ovid) for scaling up in the real-world setting.**

| **Database: Embase**  **Segment used: Embase 1974 to 2021 September 27** | | |
| --- | --- | --- |
| **Search executed: September 27^th^, 2021** | | |
| **#** | **String** | **Hits** |
| 1 | exp heart failure/ | 548275 |
| 2 | exp Telecommunications/ or exp Telemedicine/ | 89526 |
| 3 | Telehealth.mp. | 16290 |
| 4 | (mhealth or ehealth).mp. | 10557 |
| 5 | (wearable* or gadget* or sensor* or smart* or "digital" or "machine" or ("mobile" adj2 (technolog* or "app" or "apps")) or electronic* or tablet* or accelerometer* or gyroscope* or tracker or (("speech" or linguistic* or language*) adj2 (analys?s or "recognition" or monitor* or pattern*))).ti,ab. | 1270719 |
| 6 | or/2-5 | 1346543 |
| 7 | 1 and 6 | 17149 |
| 8 | (book or chapter or editorial or erratum or letter or note or short survey or tombstone or comment or conference or conference abstract or conference review).pt. | 8385097 |
| 9 | Case Study/ | 81151 |
| 10 | case report.tw. | 461920 |
| 11 | (exp animal/ or nonhuman/) not exp human/ | 6659641 |
| 12 | or/8-11 | 14707330 |
| 13 | 7 not 12 | 8231 |
| 14 | limit 13 to (adult <18 to 64 years> or aged <65+ years>) | 4515 |
| 15 | limit 14 to yr="2015 -Current" | 3032 |
| 16 | exp scale up/ | 12857 |
| 17 | (upscal* or scal* or scale up or population level or barrier* or implement* or adoption).ti,ab. | 2458938 |
| 18 | 16 or 17 | 2460734 |
| 19 | 15 and 18 | 457 |
